# Transcriptome Analysis in *Pyrus betulaefolia* Roots in Response to Short-Term Boron Deficiency

**DOI:** 10.3390/genes14040817

**Published:** 2023-03-29

**Authors:** Jing Liu, Tao Chen, Chun-Lei Wang, Xiao Liu

**Affiliations:** College of Horticulture and Landscape Architecture, Yangzhou University, Yangzhou 225009, China

**Keywords:** pear rootstock, boron deficiency, aquaporin, plant hormone, transcriptome

## Abstract

Boron (B) deficiency stress is frequently observed in pear orchards and causes a considerable loss of productivity and fruit quality. *Pyrus betulaefolia* is one of the most important rootstocks that has been widely used in pear production. The present study confirmed that the boron form of different tissues showed various changes, and the free boron content was significantly decreased under the short-term B deficiency condition. Moreover, the ABA and JA content also significantly accumulated in the root after short-term B deficiency treatment. A comprehensive transcriptome analysis of 24 h B deficiency treatment *P. betulaefolia* root was performed in this study. Transcriptome results revealed a total of 1230 up-regulated and 642 down-regulated differentially expressed genes (DEGs), respectively. B deficiency significantly increased the expression of the key aquaporin gene NIP5-1. In addition, B deficiency also increased the expression of ABA (ZEP and NCED) and JA (LOX, AOS and OPR) synthesis genes. Several MYB, WRKY, bHLH and ERF transcription factors were induced by B deficiency stress, which may relate to the regulation of B uptake and plant hormone synthesis. Overall, these findings suggested that *P. betulaefolia* root had adaptive responses to short-term B deficiency stress by improved boron absorption ability and hormone (JA and ABA) synthesis. The transcriptome analysis provided further information for understanding the mechanism of the pear rootstock responses to B deficiency stress.

## 1. Introduction

Boron (B) is an essential micronutrient for the growth of all vascular plants. More than 100 crops had been reported to suffer from B deficiency in past years [1]. Due to the narrow suitable concentration of B that can be absorbed and the low abundance of B in the soil [2,3], plants are very easily exposed to B deficiency. B plays a significant role in plant growth, including the structure and function of the cell wall, membrane integrity and carbohydrate transport and distribution [4]. B deficiency symptoms in trees can be divided into two main groups. One is the inhibition of growing points, such as the root tip, bud, flower and young leaf. The other type of symptom is the deformity of some organs, such as the shoot, leaf and fruit. Relevant anatomical studies demonstrated that B deficiency could severely damage the vascular tissues and induce hypertrophy at the tissue/cellular level [4,5,6,7]. The efficiency of B uptake, transport and utilization are closely related to B forms existing in plant tissues. Generally, B forms can be divided into two B pools: the soluble B pool (cell sap), which is a mixture of intracellular and apoplasmic fluids, and the insoluble B pool (water insoluble residue) that is mainly bound to cell wall structures. Soluble B includes free B and semi-bound B. Previous studies have suggested that the forms of B reserves may be limited to free B and semi-bound B because bound B for cell wall construction is not retranslocated [8,9]. To date, many B transporters are reported to be involved in B uptake and utilization. Generally, two types of B transporters have been identified: BORs, which perform a B export role in plant cells, and major intrinsic proteins (MIPs), including some boric acid channels. Both types of B transporters contribute to B uptake by roots, xylem loading and B distribution and utilization within the plant under B-limited conditions [4,10,11].

Acting as regulatory factors, the phytohormone is involved in the adjustment of the plant in response to nutrient stress. Many studies had reported that the phytohormone metabolism and related gene expression are associated with B deficiency. Using whole genome Affymetrix Gene Chip to give insight into the global gene expression in response to short- and long-term B deficiency in *Arabidopsis* showed that a large number of genes associated with phytohormone synthesis, transport and signal transduction were induced, especially the genes of jasmonic acid (JA), and showed the most prominent response under B deficiency [12]. Physiological and transcriptional analyses in *Brassica napus* seedlings responding to B deficiency showed that the auxin biosynthesis and efflux gene expression were affected and the indole-3-acetic acid (IAA) concentration was reduced, meanwhile, the concentration of jasmonates (JAs) and abscisic acid (ABA) were increased along with the ABA biosynthesis and sensor gene being induced to express in the shoot [13]. With the increasing concentration of B supply in *B. napus* seedlings, the concentration of ABA and IAA were reduced along with the enhancement of cytokinin (CTK) and gibberellin (GA) biosynthesis [14].

Previous studies found many transcription factors respond to B deficiency. For instance, the expression of MYB14, MYB15, MYB78, WRKY38, WRKY40 and WRKY46 genes were upregulated, while bZIP34 and bZIP61 gene expressions were lower in *Arabidopsis* plants subjected to B deprivation after 24h [15]. Transcriptome analysis found expression of bHLH and WRKY transcription factors were upregulated in the *Beta vulgaris* seedlings [16]. Kasajima et al. established that WRKY6 is a low B-induced transcription factor gene that is essential for *Arabidopsis* normal root growth under low B conditions [17]. Moreover, ERF018 is a transcription factor regulating JA synthesis, which directly binds to the promoter regions of AOC1 and AOC3. Low B promotes JA synthesis and negatively regulates the root growth of *A. thaliana* by inducing the expression of ERF018 [18].

Pear is the most important fruit in the world and the phenomenon of B deficiency often occurs in pear cultivation, greatly affecting the plant’s growth and fruit quality. Rootstocks are often used to enhance the resistance of trees and improve fruit quality and yield in pear cultivation. *P. betulaefolia* is one of the most important rootstocks that had been widely used with deep root distribution, excellent resistance to cold, waterlogging and salt. However, the response to short-term B deficiency in *P. betulaefolia* roots had not been conducted. Therefore, the investigation of responses of *P. betulaefolia* to B deficiency may provide new insights into its regulation and adaptation mechanisms. Large-scale transcriptomic analyses provided an effective tool to identify new genes that respond to the stress and biosynthesis. In this study, RNA-Seq technology was used to explore the internal changes of the molecular mechanisms of *P. betulaefolia* roots regarding the response to short-term B deficiency.

## 2. Materials and Methods

### 2.1. Plant Materials

Seeds of *P. betulaefolia* with similar size were soaked in 1% (*v/v*) sodium hypochlorite solution for 5 min and then washed three times with ultrapure water (18.25 MΩ·cm). Surface-sterilized seeds were sowed on gauze moister with ultrapure water to germination for 10 days. Thereafter, uniformly sized seedlings were sowed in nutritional substrate until three to five leaves expanded.

Subsequently, all the plants were washed with tap water to remove surface impurities after two-day starvation of tap water, and then transplanted into 15 L black plastic box (five plants per box) filled with nutrient solution. The black box had been washed with 3% hydrochloric acid (HCl) for two days and then rinsed thoroughly with deionized water before the transplantation. The composition and concentration of salt of the nutrient solution were as follows: 5 mM KNO_3_, 5 mM Ca(NO_3_)_2_·4H_2_O, 2 mM MgSO_4_·7H_2_O, 1 mM KH_2_PO_4_, 0.01 mM MnCl_2_·4H_2_O, 0.8 µM ZnSO_4_·7H_2_O, 0.3 µM CuSO_4_·5H_2_O, 0.4 µM Na_2_MoO_4_·2H_2_O, 0.02 µM FeSO_4_·7H_2_O and 0.02 µM EDTA-Na_2_. Treatment with 0.045 mM and 0 mM B were considered as control and B deficiency, respectively. All seedings were supplied with one-quarter nutrient strength and one-half nutrient-strength nutrient solution (each for 7 days) for adaptive training in sequence along with 10 min aeration to nutrient solution per day. The nutrient solution was renewed every 7 days. All plants were grown under identical conditions. The pH of all nutrient solutions was adjusted to 6.5 using 1 M NaOH. Finally, five plants from each treatment were randomly harvested after 24 h treatment.

The leaves, roots, petioles and stem (divided into stem top and stem base) were harvested separately. All samples were immediately washed up with sterile water, frozen in liquid nitrogen and stored at −80 ℃ for further analysis.

### 2.2. Analysis of Boron Forms

Different forms of B were extracted as described by Du et al. [19] with slight modifications. For each part, 3 g samples were cut into pieces of approximately 1 mm^2^, put into a plastic bottle with 15 ML of ultrapure water, and shaken at 28 °C for 24 h at 150 rpm. The sample centrifuged at 25 °C for 10 min at 10,000 rpm and then filtered through filter paper (maximum aperture: 20~25 µm). The supernatant was collected as free B. The residue was then successively extracted with 15 mL of NaCl (1 mol/L) and 15 mL of HCl (1 mol/L) solution following the same steps as above, and the supernatants were collected as semi-bound B and bound B, respectively. Three forms of B concentration of the extract were determined by the colorimetric curcumin method [19].

### 2.3. Analysis of JA and ABA

The content of ABA and JA were measured using the plant hormone abscisic acid (ABA) ELISA Kit and Plant Jasmonic acid (JA) ELISA Kit (Shanghai Enzyme-linked Biotechnology). In detail, 0.1 g root tip were thoroughly ground in liquid nitrogen and homogenized with 900 μL PBS buffer at 2–8 °C. The composition of PBS buffer was as follows: 2 mM KH_2_PO_4_, 8 mM Na_2_HPO_4_, 136 mM NaCl, 2.6 mM KCL and the pH of that was 7.4. After fully homogenizing, the sample was centrifuged at 25 °C for 20 min at 3000 rpm and the supernatant was collected to analyze the content of ABA and JA. The standard density was performed using 50 μL of different content of standard plant hormone following the manufacturer’s synopsis. For blank wells, no supernatant and HRP-conjugate reagent were added and other steps were the same as the testing sample wells. For testing sample wells, 40 μL sample dilution and 10 μL supernatant were added (the supernatant dilution is 5-fold) and gently mixed. Then, 100 μL of HRP-conjugate reagent was added to each testing sample well and incubated for 60 min at 37 °C. After that, the liquid was discarded, 700 μ L1-fold washing buffer to wash the testing sample wells was added (20-fold wash solution diluted 1-fold washing buffer with distilled water), it was allowed to stand for 30 s and the washing buffer was discarded. The step of washing was repeated 5 times. Then, 50 μL Chromogen Solution A and B was added and preserved for 15 min at 37 °C under no light conditions. Finally, 50 μL of stop solution was added to stop the reaction and the absorbance was read at 450 nm within 15 min.

### 2.4. RNA-Seq Analisis

#### 2.4.1. Library Preparation for RNA Sequencing

To gain insight into root cell transcriptomic dynamics, RNA-seq analyses was performed using root tips collected from the control roots and 24 h B deficiency roots in triplicates. Total RNA of each sample was extracted using the Trizol kit (Promega, MA, USA) following the manufacturer’s synopsis. A total amount of 1 μg RNA per sample was used as input material for the RNA sample preparations. Sequencing libraries were generated using NEBNext^®^ Ultra™ RNA Library Prep Kit for Illumina^®^ following the manufacturer’s recommendations, and index codes were added to attribute sequences to each sample. Briefly, mRNA was purified from total RNA using poly-T oligo-attached magnetic beads. Fragmentation was carried out using divalent cations under elevated temperature in NEBNext First Strand Synthesis Reaction Buffer (5X). First strand cDNA was synthesized using random hexamer primer and RNase H. Second strand cDNA synthesis was subsequently performed using buffer, dNTPs, DNA polymerase I and RNase H. The library fragments were purified with QiaQuick PCR kits and elution with EB buffer, then terminal repair, A-tailing and adapter added were implemented. The aimed products were retrieved, and PCR was performed, then the library was completed.

#### 2.4.2. Library Clustering and Sequencing

The clustering of the index-coded samples was performed on a cBot cluster generation system using HiSeq PE Cluster Kit v4-cBot-HS (Illumina) according to the manufacturer’s instructions. After cluster generation, the libraries were sequenced on an Illumina platform by Benagen Technology Co., Ltd., Wuhan, China and 150 bp paired-end reads were generated.

#### 2.4.3. RNA-Seq Data Analysis

The raw data were first processed with FastQC (Version: 0.11.9, default) to filter out adapters and low-quality sequences. Then, the clean reads were mapped to the *Pyrus bretschneideri* genome (https://www.ncbi.nlm.nih.gov/genome/12793) using Star (Version: 2.7.9a, default). In order to obtain the comprehensive functional information of the genes, we also annotated the gene functions of uniprot database by BlastP. RSEM (Version: 1.3.3, default) was used to calculate gene expression level for each sample expressed as fragments per kilobase of transcript per million fragments mapped (FPKM). The gene expression levels in stressed samples were compared with those in control samples in order to identify the differentially expressed genes (DEG) by DESeq2 (Version: 1.34.0, default). The DEGs were detected as previously described based on the parameters: |log_2_ ^foldchange^| ≥ 1 and corrected *p* < 0.05 based on the three biological replicates. Gene Ontology (GO) and Kyoto Encyclopedia of Genes and Genomes (KEGG) enrichment analyses for the DEGs were performed using the clusterProfiler version 3.8. All RNA-seq data have been uploaded to the National Center for Biotechnology Information (NCBI) Gene Expression Omnibus (GEO) database (GEO accession number: GSE218373). All DEG data were listed in Table S1.

### 2.5. cDNA Synthesis and Quantitative Real-Time (qRT)-PCR Analysis

First-strand cDNA was synthesized using the HiScript^®^ III RT SuperMix for qPCR (+gDNA wiper) (Vazyme Biotechnolog, Nanjing, China). To validate the reliability of RNA-seq data, several genes were randomly selected for qRT-PCR analysis. qRT-PCR was determined with a Bio-Rad CFX96 instrument (Bio-Rad, Waltham, MA, USA) by using ChamQ SYBR qPCR Master Mix (Vazyme Biotechnology, Nanjing, China). In order to validate the accuracy of DEGs, the DEGs from the aquaporins and hormone metabolism were analyzed by qRT-PCR. Gene-specific primers were designed using the software Primer5 (Table S2), and the specificity and quality of each primer pair were checked by melting curve analysis and sequencing. The amplification reactions were as follows: a pre-denaturation step of 95 °C for 3 min, 40 cycles of 95 °C for 5 s and 65 °C for 5 s and a melting curve analysis according to Vazyme Biotechnology’s analysis instructions. The Livak [20] method was used to calculate gene relative expression levels.

### 2.6. Statistical Analysis

Error bars indicate standard error (SE) of the means. Data were compared by one-way analysis of variance (SPSS for Windows 17.0; SPSS Inc., Chicago, IL, USA). Differences were determined by Tukey test (*p* < 0.05) and indicated by different letters. Bar graphs were drawn using the GraphPad Prism 7.0 scientific software (San Diego, CA, USA).

## 3. Results

### 3.1. Subsection

#### 3.1.1. Boron Concentration in Various Plant Parts under the Short B Deficiency Treatment

**Figure 1:**
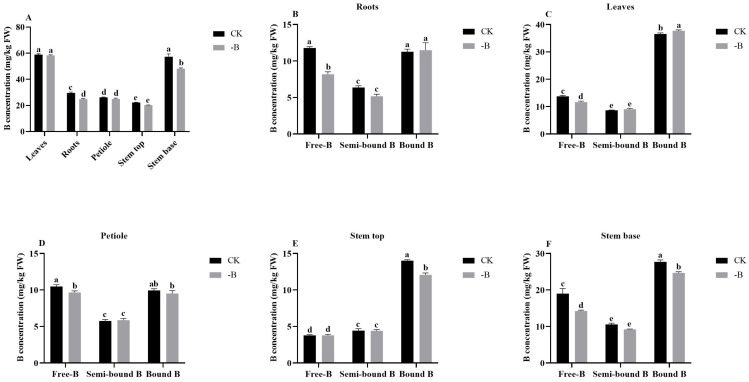
Total B concentration in *P. betulaefolia* under B-deficient conditions. (**A**) Total B concentration in different tissues. (**B**–**F**) refer to three B forms in different tissues. Values are means of three replicates ± SE. Bars with different letters indicate significant differences for the same B level at *p* < 0.05.

After 24 h B deficiency treatment, the concentration of total B in roots and stem base were significantly decreased, whereas the leaves, petiole and stem top showed no remarkable differences (Figure 1A). In detail, the free B concentration in roots, leaves, petiole and stem base were significantly decreased, whereas there were no remarkable differences in the stem top (Figure 1B–E). Moreover, the bound B concentration significantly increased in leaves and decreased in stem top and stem base, respectively (Figure 1C,E,F). Semi-bound B concentration had no significant differences in all plant parts (Figure 1).

#### 3.1.2. Analysis the Content of JA and ABA in Root Tips under the Short B Deficiency Treatment

**Figure 2:**
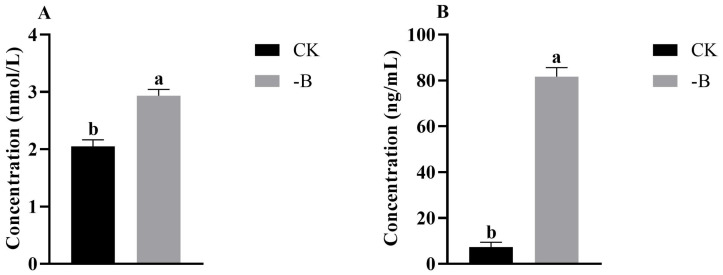
JA (**A**) and ABA (**B**) content in *P. betulaefolia* root under B-deficient conditions. Values are means of three replicates ± SE. Bars with different letters indicate significant differences at *p* < 0.05.

After short-term treatment of B deficiency, the content of JA and ABA was significantly increased in root tips (Figure 2).

#### 3.1.3. RNA-Seq Transcriptome and Analysis of Differentially Expressed Genes

**Figure 3:**
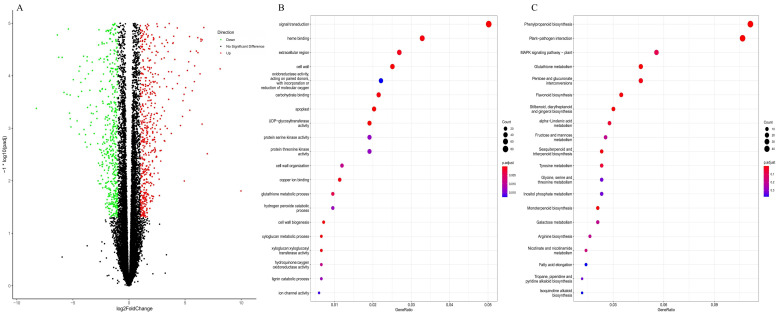
Transcriptome sequencing analysis of different expressed genes (DEGs) in *P. betulaefolia* root under 24 h B-deficiency treatment. (**A**) refer to volcano plot of DEGs; (**B**,**C**) refer to GO and KEGG analysis.

Considering the free B concentration was sharply decreased in roots, RNA-Seq libraries were established from the root tips after 24 h B deficiency treatment. RNA-Seq generated more than 36.04 million raw reads for each sample. After quality control, a larger number of clean reads from 35.81 to 39.81 million were generated and the Q30 percentage was over 93.00% (Table 1). Among all the libraries, the GC content was approximately 46.50%, and 91.39–91.90% of unique reads were mapped to the *P. bretschneideri* genome (Table 1). A total of 1230 genes in B deficiency root tips were up-regulated and 642 genes were down-regulated (Figure 3A).

All DEGs from the libraries were subjected to GO functional annotation and KEGG pathway enrichment analysis. In the GO database, signal transduction was the most enriched in the “biological process” category; cell wall and apoplast were the most enriched in the “cellular component” category; and heme binding and oxidoreductase were the most enriched in the “molecular function” category (Figure 3B). In the KEGG pathway analysis, the DEGs were involved in five enriched pathways: phenylpropanoid biosynthesis, plant–pathogen interaction, MAPK signaling pathway-plant, glutathione metabolism, and pentose and glucuronate interconversions (Figure 3C).

To confirm the accuracy of the libraries, DEGs from Table 2, Table 3 and Table 4 were selected to analyze expression by qRT-PCR. The result of qRT-PCR analysis showed the expression of DEGs were consistent with that obtained from high-throughput sequencing (Figure S1).

#### 3.1.4. The Expression of Aquaporin Genes under B Deficiency

As shown in Table 2, several TIP and NIP genes were identified, the expression of TIP4-1-like (LOC103937833) and NIP5-1 (LOC103962656) were significantly up-regulated.

#### 3.1.5. The Gene Expression in ABA Biosynthesis Pathway under B Deficiency

**Figure 4:**
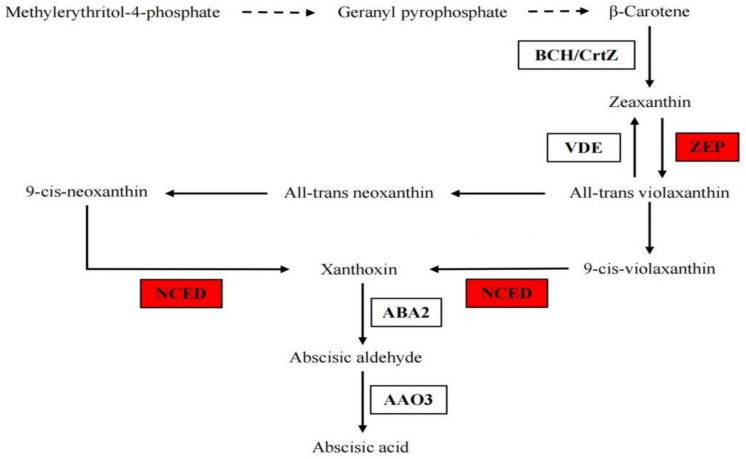
Differentially expressed genes involved in ABA synthesis. Red refers to the significantly up-regulated genes. BCH/CrtZ (β–Carotene hydroxylase); ZEP (Zeaxanthin epoxidase); VDE (Violaxanthin de-epoxidase); NCED (9–cis–epoxycarotenoid dioxygenase); ABA2 (Xanthoxin dehydrogenase); AAO (Abscisic-aldehyde oxidase).

After short B deficiency treatment, the gene expression of two ZEP (LOC103937745 and LOC103945929) and one NCED (LOC103957334) genes were up-regulated (Figure 4, Table 3). The expression of BCH, VDE, ABA2 and AAO genes had no significant changes after B deficiency treatment.

#### 3.1.6. The Gene Expression in JA Biosynthesis Pathway under B Deficiency

**Figure 5:**
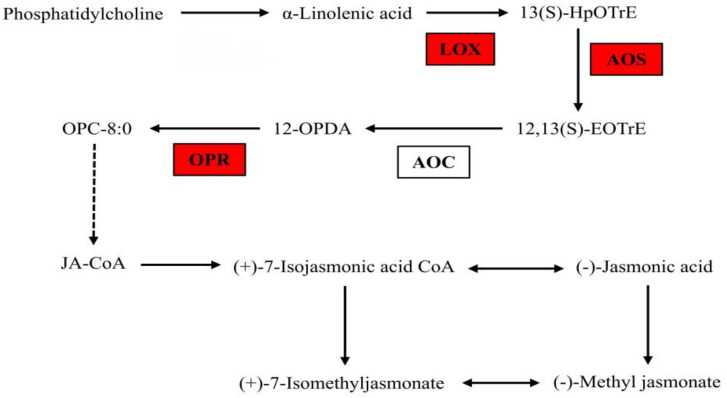
Differentially expressed genes involved in JA synthesis. Red refers to the significantly up-regulated genes. LOX (lipoxygenase); AOS (allene oxide synthase); AOC (allene oxide cyclase); OPR (12−oxophytodienoic acid reductase).

The gene expression of LOX, AOS and OPR, which are involved in the JA biosynthesis pathway, were changed after short B deficiency treatment. In detail, the expression of LOX 3-1 (LOC103941411), LOX 3-1-like (LOC125471779), AOS 3-like (LOC103942464) and OPR 11 (LOC103952517) were up-regulated (Figure 5, Table 4). The expression of AOC genes had no significant changes after B deficiency treatment.

#### 3.1.7. The Differentially Expressed Transcription Factor Genes Response to Short Term B Deficiency

As showed in Table 5, the transcription factor genes of 9 MYB genes (LOC103953105, LOC103941953, LOC103946050, LOC103937539, LOC103959532, LOC125472789, LOC125477110, LOC103944477 and LOC103931986), 14 WRKY genes (LOC103958164, LOC103952502, LOC103964384, LOC103943771, LOC103940628, LOC125473081, LOC125469162, LOC103953687, LOC103956875, LOC103951248, LOC103945359, LOC103953663, LOC125468857 and LOC103941856), 5 bHLH genes (LOC103929974, LOC103941211, LOC103945773, LOC103958789 and LOC125475930) and 10 ERF genes (LOC103947947, LOC103950248, LOC103955515, LOC103932979, LOC103929680, LOC103928186, LOC103941900, LOC103935996, LOC103959790 and LOC103953878) were significantly up-regulated under the short B deficiency treatment.

## 4. Discussion

Boron (B) is an important micronutrient for higher plants, and B deficiency is a worldwide nutrition problem [1]. Previous studies have shown that B deficiency severely alters plant morphology and physiology processes. B deficiency inhibits the root growth and leaf expansion and affects photosynthesis, carbohydrate metabolism and protein synthesis, influences the stabilization of the cell membrane and cell wall and decreases the pectin content [2]. B forms can be divided into three B pools: free B, which exists extracellularly, semi-bound B that is mainly combined with entocyte, and bound B that is mainly bound to cell wall structures [8]. In this study, we found the leaves contain more bound B than other parts because of a higher pectin content that combined with B which involved in cell wall construction in leaves. The roots, stem base and petiole have high free B due to these organs are the main part of B absorption, transport and allocation, respectively. In whole plant, the bound B has the highest concentration (more than 50%) and followed by free B and semi-bound B. Moreover, Wang et al. suggest that B efficient rootstock enhanced the transport of available B (free B and semi-bound B) into leaves, and that scion employed more available B to be involved in metabolism and to synthesize the cell wall under B-limited conditions [8]. In the current study, the concentration of free B was significantly decreased in roots after short B deficiency treatment, it suggested that the roots are the most sensitive to B deficiency, and free B content decreased first while compared with other B forms, the free B may transport to the aboveground organization.

Roots uptake B from environment and transport it to the aerial part. The process of uptake can take place passively under B efficiency or through active transport under B deficiency. Belonging to the major intrinsic protein families (MIPs), aquaporins are a kind of specific protein that can mediate the passive diffusion of water and the transport of some small molecules [21,22]. NIP5-1, which belongs to NOD26-like intrinsic proteins, which is a kind of aquaporin, had been confirmed to be involved in B absorption. The *AtNIP5-1* localizes in the epidermis, cortex and endodermis in roots, which are over expressed in the apical elongation zones and root hair regions under B deficiency to enhance B absorption. Mutants of *AtNIP5-1* showed a decrease in B absorption, suggesting the *AtNIP5-1* is a major B absorption channel [23,24]. As shown in Table 2, we found the *NIP5-1* expression was up-regulated after 24 h B deficiency, suggesting the *NIP5-1* in *P. betulaefolia* may play an important role in B absorption under B deficiency. To date, some regulated B transport-related genes have been reported in plants. For instance, the expression of WRKY transcription factors was up-regulated, which is consist with the expression of NIP5;1 in the *B. vulgaris* seedlings under the B deficiency condition [16]. Low B induced the promoter activity of WRKY6 around the root tip, and functional loss of WRKY6 decreased root elongation under B deficiency in *Arabidopsis* [17]. In *B. napus*, transcription factor *BnaA9.WRKY47* contributes to the adaptation of *B. napus* to low boron stress by up-regulating the boric acid channel gene *BnaA3.NIP5-1* [25]. Moreover, Feng et al. found that repression of transcription factor *AtWRKY47* confers tolerance to boron toxicity in *A. thaliana* [26]. In this study, some *PbWRKYs* were identified; however, whether they can regulate *PbNIP5-1* expression to improve the B uptake in pear rootstock needs further study in the future.

A change in phytohormones is one of the most significantly adaptive strategies used by plants in their response to adverse conditions. Levels of JA often increase following exposure to a number of biotic and abiotic stresses, which is consistent with the role of JA in mediating various defense responses [27]. Jasmonate-mediated growth inhibition promotes plant adaptation to B deficiency. Transcriptomic studies have revealed that B deficiency activates the transcription of JA biosynthesis and signaling genes in *Arabidopsis* [12], *B. napus* [13] and *Pisum sativum* [28]. The JA signaling pathway is activated in response to the accumulation of ROS under B deficiency stress [12]. Several sources of oxidative stress stimulate JA accumulation and JA signaling, which are considered to be involved in the containment of lesions that form in response to ROS [12]. Moreover, ABA is a major player in mediating plant adaptation to stress and is induced by a number of abiotic stresses. Peng et al. also found the key enzyme genes ZEP and NCED were significantly induced by boron deficiency [12]. ABA can accelerate cell senescence, thereby promoting nutrient recycling, which relieves the inhibition of boron deficiency on plant growth. In the current study, some JA and ABA synthesis genes were also up-regulated by B deficiency, which is consistent with the increased content of JA and ABA. Additionally, Gomez-Soto reported that ABA treatment increased *AtNIP5-1* activity and induced B uptake [29]. Taken together, it suggests that *P. betulaefolia* may improve its adaptive ability to boron deficiency by increasing JA and ABA synthesis to promote boron uptake and relieve ROS damage.

## Figures and Tables

**Table 1:** Summary of transcriptome sequencing data of all libraries. B1–B3 refer to three B-deficient replicates; CK1-CK3 refer to three B-adequate replicates.

Samples	Raw Reads	Clean Reads	Q20 (%)	Q30 (%)	GC Content (%)	Total Map
B1	36,056,316	35,817,088	97.48	92.83	46.70	19,854,248 (91.74%)
B2	36,047,720	35,884,386	97.93	93.82	46.90	19,901,778 (91.90%)
B3	40,023,668	39,819,658	97.98	93.99	46.70	19,905,043 (91.79%)
CK1	39,896,460	39,708,496	97.92	93.81	46.65	17,908,544 (91.39%)
CK2	40,025,276	39,803,556	97.67	93.25	46.67	17,942,193 (91.83%)
CK3	40,046,248	39,810,086	97.75	93.42	46.67	19,909,829 (91.57%)

**Table 2:** Expression levels of aquaporin genes.

Gene Description	Log_2_^Fold Change^	ID
TIP	2.18	LOC103937833
	0.11	LOC103930013
	−0.48	LOC103930703
	−0.24	LOC103938012
	0.10	LOC103941273
	0.34	LOC103944840
	−0.14	LOC103948949
	0.26	LOC103957101
	0.60	LOC103966432
NIP	2.60	LOC103962656
	−0.72	LOC103944046
	0.67	LOC103950753
	−0.34	LOC125477097

**Table 3:** Expression levels of ABA synthesis genes. NE refer to not expressed.

Gene Description	Log_2_^Fold Change^	ID
ZEP	1.44	LOC103937745
	1.17	LOC103945929
	−0.02	LOC103956812
	NE	LOC125470765
NCED	1.55	LOC103957334
	−0.16	LOC125476311
	−0.15	LOC103935979
	NE	LOC103955326
BCH	−0.14	LOC103958212
	0.34	LOC103964324
	NE	LOC103953392
VDE	0.11	LOC103968091
ABA2	−0.17	LOC103957656
	−0.60	LOC125478256
	NE	LOC103945571
	NE	LOC125478257
AAO	−0.75	LOC103937039
	ND	LOC103929500

**Table 4:** Expression levels of JA synthesis genes. NE refer to not expressed.

Gene Description	Log_2_^Fold Change^	ID
LOX	1.23	LOC103941411
	1.24	LOC125471779
	NE	LOC103931981
	NE	LOC103944208
OPR	2.69	LOC103952517
	−0.64	LOC103951930
	0.23	LOC103952512
	−0.74	LOC103952514
	0.06	LOC103952519
	−0.14	LOC103952523
	−0.21	LOC125478814
AOS	−0.19	LOC103954131
	0.10	LOC103955456
	2.44	LOC103942464
	NE	LOC103930300
AOC	0.07	LOC103929185
	−0.17	LOC103947146
	−0.93	LOC103947801

**Table 5:** Expression levels of transcription factor genes.

Function	Log_2_^Fold Change^	ID
MYB	3.53	LOC103953105
	2.58	LOC103941953
	1.65	LOC103946050
	1.61	LOC103937539
	1.49	LOC103959532
	1.47	LOC125472789
	1.24	LOC125477110
	1.19	LOC103944477
	1.02	LOC103931986
WRKY	3.52	LOC103958164
	3.37	LOC103952502
	2.59	LOC103964384
	2.56	LOC103943771
	2.08	LOC103940628
	1.93	LOC125473081
	1.58	LOC125469162
	1.55	LOC103953687
	1.53	LOC103956875
	1.35	LOC103951248
	1.33	LOC103945359
	1.31	LOC103953663
	1.15	LOC125468857
	1.06	LOC103941856
bHLH	2.20	LOC103929974
	1.28	LOC103941211
	1.55	LOC103945773
	2.69	LOC103958789
	1.29	LOC125475930
ERF	3.90	LOC103947947
	3.54	LOC103950248
	3.26	LOC103955515
	2.84	LOC103932979
	2.83	LOC103929680
	2.35	LOC103928186
	2.22	LOC103941900
	2.13	LOC103935996
	1.63	LOC103959790
	1.28	LOC103953878

## Data Availability

Transcriptome data were published on NCBI (Accession number: GSE218373).

## Supplementary Materials

The following supporting information can be downloaded at: https://www.mdpi.com/article/10.3390/genes14040817/s1. Figure S1: qRT-PCR validation of DEGs; Table S1: List of all DEGs; Table S2: List of primers.

## References

[1] Rerkasem B., Jamjod S., Pusadee T. (2020). Productivity limiting impacts of boron deficiency, a review. Plant Soil.

[2] Reid R. (2014). Understanding the boron transport network in plants. Plant Soil.

[3] Shireen F., Nawaz M.A., Chen C., Zhang Q.K., Zheng Z.H., Sohail H., Sun J.Y., Cao H.S., Huang Y., Bie Z.L. (2018). Boron: Functions and Approaches to Enhance Its Availability in Plants for Sustainable Agriculture. Int. J. Mol. Sci..

[4] Wang N.N., Yang C.Q., Pan Z.Y., Liu Y.Z., Peng S.A. (2015). Boron deficiency in woody plants: Various responses and tolerance mechanisms. Front. Plant Sci..

[5] Yang C.Q., Liu Y.Z., An J.C., Li S., Jin L.F., Zhou G.F., Wei Q.J., Yan H.Q., Wang N.N., Fu L.N. (2013). Digital Gene Expression Analysis of Corky Split Vein Caused by Boron Deficiency in ‘Newhall’ Navel Orange (*Citrus sinensis* Osbeck) for Selecting Differentially Expressed Genes Related to Vascular Hypertrophy. PLoS ONE.

[6] Sutinen S., Vuorinen M., Rikala R. (2006). Developmental disorders in buds and needles of mature Norway spruce, *Picea abies* (L.) Karst., in relation to needle boron concentrations. Trees-Struct. Funct..

[7] Mei L., Li Q.H., Wang H., Sheng O., Peng S.A. (2016). Boron deficiency affects root vessel anatomy and mineral nutrient allocation of *Poncirus trifoliata* (L.) Raf. Acta Physiol. Plant..

[8] Wang N.N., Yan T.S., Fu L.N., Zhou G.F., Liu Y.Z., Peng S.A. (2014). Differences in boron distribution and forms in four citrus scion-rootstock combinations with contrasting boron efficiency under boron-deficient conditions. Trees-Struct. Funct..

[9] Liu G.D., Jiang C.C., Wang Y.H. (2011). Distribution of boron and its forms in young “Newhall” navel orange (*Citrus sinensis* Osb.) plants grafted on two rootstocks in response to deficient and excessive boron. Soil Sci. Plant Nutr..

[10] Hanaoka H., Uraguchi S., Takano J., Tanaka M., Fujiwara T. (2014). *OsNIP3;1*, a rice boric acid channel, regulates boron distribution and is essential for growth under boron-deficient conditions. Plant J..

[11] Miwa K., Takano J., Fujiwara T. (2006). Improvement of seed yields under boron-limiting conditions through overexpression of BOR1, a boron transporter for xylem loading, in *Arabidopsis thaliana*. Plant J..

[12] Peng L.S., Zeng C.Y., Shi L., Cai H.M., Xu F.S. (2012). Transcriptional Profiling Reveals Adaptive Responses to Boron Deficiency Stress in *Arabidopsis*. Z. Fur Nat. Sect. C-A J. Biosci..

[13] Zhou T., Hua Y.P., Huang Y.P., Ding G.D., Shi L., Xu F.S. (2016). Physiological and Transcriptional Analyses Reveal Differential Phytohormone Responses to Boron Deficiency in *Brassica napus* Genotypes. Front. Plant Sci..

[14] Eggert K., von Wiren N. (2017). Response of the plant hormone network to boron deficiency. New Phytol..

[15] Gonzalez-Fontes A., Rexach J., Quiles-Pando C., Begona Herrera-Rodriguez M., Camacho-Cristobal J.J., Teresa Navarro-Gochicoa M. (2013). Transcription factors as potential participants in the signal transduction pathway of boron deficiency. Plant Signal. Behav..

[16] Song X., Wang X., Song B., Wu Z., Zhao X., Huang W., Riaz M. (2021). Transcriptome analysis reveals the molecular mechanism of boron deficiency tolerance in leaves of boron-efficient *Beta vulgaris* seedlings. Plant Physiol. Biochem..

[17] Kasajima I., Ide Y., Hirai M.Y., Fujiwara T. (2010). WRKY6 is involved in the response to boron deficiency in *Arabidopsis thaliana*. Physiol. Plant..

[18] Huang Y.P., Wang S.L., Wang C., Ding G.D., Cai H.M., Shi L., Xu F.S. (2021). Induction of jasmonic acid biosynthetic genes inhibits *Arabidopsis* growth in response to low boron. J. Integr. Plant Biol..

[19] Du C.W., Wang Y.H., Xu F.S., Yang Y.H., Wang H.Y. (2002). Study on the physiological mechanism of boron utilization efficiency in rape cultivars. J. Plant Nutr..

[20] Livak K.J., Schmittgen T.D. (2001). Analysis of relative gene expression data using real-time quantitative PCR and the 2(T)(-Delta Delta C) method. Methods.

[21] Tanaka M., Fujiwara T. (2008). Physiological roles and transport mechanisms of boron: Perspectives from plants. Pflug. Arch.-Eur. J. Physiol..

[22] Li G.W., Santoni V., Maurel C. (2014). Plant aquaporins: Roles in plant physiology. Biochim. Biophys. Acta-Gen. Subj..

[23] Takano J., Wada M., Ludewig U., Schaaf G., von Wiren N., Fujiwara T. (2006). The Arabidopsis major intrinsic protein NIP5;1 is essential for efficient boron uptake and plant development under boron limitation. Plant Cell.

[24] Wang S.L., Yoshinari A., Shimada T., Hara-Nishimura I., Mitani-Ueno N., Ma J.F., Naito S., Takano J. (2017). Polar Localization of the NIP5;1 Boric Acid Channel Is Maintained by Endocytosis and Facilitates Boron Transport in *Arabidopsis* Roots. Plant Cell.

[25] Feng Y.N., Cui R., Wang S.L., He M.L., Hua Y.P., Shi L., Ye X.S., Xu F.S. (2020). Transcription factor *BnaA9.WRKY47* contributes to the adaptation of *Brassica napus* to low boron stress by up-regulating the boric acid channel gene *BnaA3.NIP5;1*. Plant Biotechnol. J..

[26] Feng Y.N., Cui R., Huang Y.P., Shi L., Wang S.L., Xu F.S. (2021). Repression of transcription factor *AtWRKY47* confers tolerance to boron toxicity in *Arabidopsis thaliana*. Ecotoxicol. Environ. Saf..

[27] Noriega G., Cruz D.S., Batlle A., Tomaro M., Balestrasse K. (2012). Heme Oxygenase is Involved in the Protection Exerted by Jasmonic Acid Against Cadmium Stress in Soybean Roots. J. Plant Growth Regul..

[28] Chen X., Humphreys J.L., Ru Y.Q., He Y.T., Wu F.H., Mai J.W., Li M., Li Y.L., Shabala S., Yu M. (2022). Jasmonate signaling and remodeling of cell wall metabolism induced by boron deficiency in pea shoots. Environ. Exp. Bot..

[29] Gomez-Soto D., Galvan S., Rosales E., Bienert P., Abreu I., Bonilla I., Bolanos L., Reguera M. (2019). Insights into the role of phytohormones regulating p*AtNIP5;1* activity and boron transport in *Arabidopsis thaliana*. Plant Sci..

