# Supplementary figures and images for: Transcriptome Analysis in Pyrus betulaefolia Roots in Response to Short-Term Boron Deficiency
Source: Genes (Basel). 2023 Mar 29;14(4):817. doi: 10.3390/genes14040817 (PMC10137548; doi:10.3390/genes14040817)

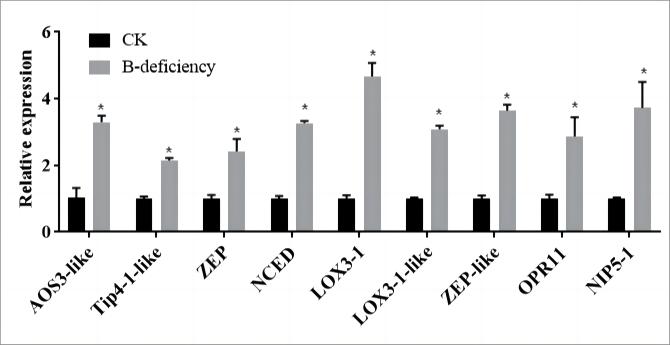

Supplement: Supplementary file 1 [file genes-14-00817-s001.zip › genes-2223790-supplementary Figure S1.jpg]
